# Formononetin improved healthspan *via* the P53/P21/CDK4 pathway in SAMP8 mice and DAF-16/SKN-1 signaling pathway in *C.elegans*

**DOI:** 10.3389/fphar.2026.1918524

**Published:** 2026-09-23

**Authors:** Bo Liu, Di Cui, Jing Liu, Yunyan Xu

**Affiliations:** Key Lab for Basic Pharmacology and Joint International Laboratory of Ethnomedicine of Ministry of Education, Zunyi Medical University, Zunyi, China

**Keywords:** C.elegans, DNA methylation, Formononetin, healthy aging, oxidative stress, SAMP8 mice

## Abstract

**Objective:**

To investigate the healthy aging effects of Formononetin and its underlying molecular mechanisms.

**Methods:**

SAMP8 mice were administered Formononetin (4, 8, 16 mg/kg, po) for 5 months. Behavioral tests were performed in 9-month-old mice, followed by histopathology. 5-methylcytosine (5-mC) level in the brain were detected by immunofluorescence, aging-related protein expression was measured by Western blot. In *C*. *elegans*, the lifespan, stress resistance, and aging-related gene expressions were assessed. Mutant strains (*daf-16* and *skn-1*) were used to verify pathway dependency.

**Results:**

Formononetin dose-dependently ameliorated cognitive deficits, anxiety-like behaviors, and motor decline in SAMP8 mice, attenuated neuronal damage in the hippocampal CA1, DG and cerebral cortex, reversed 5-mC level, downregulated protein expression of P53, P21, while upregulated CDK4 and Nrf2. In *C*. *elegans*, Formononetin (37.30 μmol/L) significantly extended lifespan under normal, thermal stress, and oxidative stress conditions, improved locomotion, reduced lipofuscin accumulation, increased total SOD activity, and upregulated *skn-1, sir-2.1, daf-16* and *sod-3* mRNA. No lifespan extension was observed in daf-16 or skn-1 mutants.

**Conclusion:**

Formononetin effectively attenuated cognitive deficits, ameliorated neuronal damage and increased 5-mC level, possibly *via* regulated P53, P21, CDK4 and Nrf2 protein expression in the brain of SAMP8 mice. In *C. elegans*, Formononetin extended lifespan, increased stress resistance, and improved locomotion and lipofuscin accumulation, possibly *via* regulated expression of DAF-16 and SKN-1.

## 1 Introduction

With the acceleration of global population aging, the incidence of aging-related diseases continues to rise, imposing a heavy burden on healthcare systems worldwide. Aging is a complex, multifactorial biological process characterized by genomic instability, telomere attrition, epigenetic alterations, loss of proteostasis, disabled macroautophagy, deregulated nutrient-sensing, mitochondrial dysfunction, cellular senescence, stem cell exhaustion, altered intercellular communication, chronic inflammation, and dysbiosis (Kroemer et al., 2025; López-Otin et al., 2023). Brain aging is of particular concern due to its close association with cognitive decline and the development of neurodegenerative disorders such as Alzheimer’s disease (Jin et al., 2024). The search for safe and effective healthy aging interventions have become a major focus of biomedical research.

Natural products offer unique advantages in healthy aging drug discovery due to their multi-targeting properties and low toxicity (Newman and Cragg, 2020). Formononetin (FMN), an isoflavone derived from plants such as *Astragalus membranaceus* and red clover, exhibits diverse pharmacological activities including antioxidant, anti-inflammatory, neuroprotective, and anti-tumor effects (Tay et al., 2019). Formononetin has good pharmacokinetics, with rapid oral absorption, a maximum plasma concentration between 30 and 60 min, and a half-life of approximately 2 h (Kim et al., 2022; Sharma and Kabra, 2025). Recent studies have shown that FMN can alleviate oxidative stress injury by activating the Nrf2/ARE pathway (Wang et al., 2024) and exert protective effects in various neurodegenerative disease models (Liu et al., 2024; Pingale and Gupta, 2025; Sharma and Kabra, 2025). However, whether FMN possesses systemic healthy aging effect, as well as its underlying molecular mechanism, remain largely unknown.

The SAMP8 (Senescence Accelerated Mouse Prone 8) mouse is a well-established accelerated aging model characterized by early-onset cognitive impairment, learning and memory deficits, anxiety-like behavior, and neuronal damage, and has been widely used in aging and neurodegenerative disease research (Liu et al., 2020a; Liu et al., 2020b; Liu et al., 2023). The SAMP8 mouse model is an excellent model used to verify the conservation of this pathway in the mammalian system, and to specifically evaluate the improvement effect of intervention on aging-related cognitive decline as well as the protective effect on neuropathology (Liu et al., 2020b). The nematode *Caenorhabditis elegans* (*C*. *elegans*), with its short lifespan, genetic tractability, and highly conserved aging-related signaling pathways, serves as an ideal model for anti-aging drug screening and mechanistic studies (Shen et al., 2018). The mutual confirmation of the conclusions drawn from these two evolutionarily distant models will greatly enhance the robustness of the research results and the credibility of their application in human aging intervention (Yu et al., 2025a).

In this study, we employed a combined strategy using SAMP8 mice and *C. elegans* to systematically evaluate the healthy aging effect of FMN. We further elucidated its mechanisms from multiple perspectives, including behavioral assessment, neuropathology, epigenetics, cell cycle regulation, and antioxidant signaling pathways. Our findings provide a theoretical basis for the development of FMN as a potential healthy aging agent.

## 2 Materials and methods

### 2.1 Mouse experiment

#### 2.1.1 Animals

Four-month-old male SAMP8 and SAMR1 mice were obtained from the Experimental Animal Center of Peking University (license no. SCXK2016-0010). Mice were housed under specific pathogen-free (SPF) conditions in the animal facility of the Key Laboratory of Basic Pharmacology, Zunyi Medical University (license no. SYXK 2014–0003). The housing environment was maintained under a standardized 12 h light/dark cycle, with ambient temperature controlled at 22 °C–25 °C and relative humidity at 50%–70%. Mice received *ad libitum* access to autoclaved tap water and standard rodent chow. All experimental procedures were conducted in strict accordance with the “Guidelines on the Humane Treatment of Laboratory Animals” issued by the Ministry of Science and Technology of the People’s Republic of China (2006), and the study protocol was prospectively reviewed and approved by the Institutional Animal Care and Use Committee of Zunyi Medical University (approval no. [2020]2–142).

#### 2.1.2 Drugs

Formononetin (FMN, C_16_H_12_O_4_; molecular weight: 268.26; purity ≥98%) was purchased from Shanghai Jizhi Biocmical Technology Co., Ltd. (Shanghai, China). For the treatment of mice, FMN was dissolved in normal saline (NS) to form a suspension, and diluted to the concentration of 4 mg/kg, 8 mg/kg and 16 mg/kg, which were used in animal experiments. FMN was dissolved in DMSO and was used in *C. elegans* experiments. All reagents were of analytical-reagent grade and were commercially available.

#### 2.1.3 Mouse experimental design

Male mice were randomly divided to SAMR1, SAMP8, SAMP8+FMN (4 mg/kg), SAMP8+FMN (8 mg/kg), SAMP8+FMN (16 mg/kg) groups, 10 in each group. The administration group were given FMN by gavage for 5 months starting at the 4-month-old of age. The dosage of the FMN used in mice is based on our previous research (Liu et al., 2024). Meanwhile, the SAMR1 and SAMP8 groups received NS. Behavioral experiments were performed at 9 months of age. After behavioral tests, the mice were euthanatized, and the brains of five mice in each group were fixed for pathological examination and the hippocampus of remaining brains were frozen at 80 °C for other analysis.

#### 2.1.4 Behavioral tests

##### 2.1.4.1 Y maze

Animals were allowed to explore the maze for 10 min and the sequence of the arm entries and total numbers of arm choices were monitored and recorded by a camcorder (Topscan, CleverSys, Inc., Reston, VA, USA). The correct rate of spontaneous alternation was calculated by the formula as [number of alternation/(number of total armentries-2)] × 100% (Liu et al., 2020a). All video analyses were performed in a blinded manner.

##### 2.1.4.2 Novel object recognition test (NOR)

The NOR test is composed of a reaction box (width of 40 cm × height of 40 cm × depth of 45 cm), camera equipment and behavioral analysis software (Topscan, CleverSys, Inc., Reston, VA, USA). The experimental procedures are consistent with our previous study (Liu et al., 2024; Liu et al., 2020a). All of the mice were free to explore in the reaction box for 5 min. The distance between the mouse’s nose and the novel blue cone within 2 cm was defined as exploratory behavior. The novel blue cone exploration time were recorded. All video analyses were performed in a blinded manner.

##### 2.1.4.3 The elevated plus maze (EPM)

Mice were put in the central area and the head toward the open arm and allowed to explore freely for 10 min, The Top View Animal Behavior Analyzing System (Version 3.00, CleverSys Inc, USA) was used to record the duration time in open arms. The arms were cleaned with 70% alcohol before the test of each animal. The time spent in the open arms were calculated as previous study (Liu et al., 2024). All video analyses were performed in a blinded manner.

##### 2.1.4.4 Open field test (OFT)

The cuboid box was cleaned with 70% alcohol before the test of another animal. The mice were habituated to the experimental environment for 30 min. Afterwards, mice were placed in the central location, and the Top View Animal Behavior Analyzing System (Version 3.00, CleverSys Inc, USA) started to record movement for 10 min. The time spent in center area were recorded (Liu et al., 2023). All video analyses were performed in a blinded manner.

#### 2.1.5 Nissl staining

Mice were anesthetized after behavioral testing, and brains were perfused with ice-cold phosphate buffered saline (PBS), followed by 4% paraformaldehyde through the heart. Then, the brain tissue was embedded and sectioned. The paraffin-embedded sections of the brain were deparaffinized and rehydrated in xylene and gradient alcohol solutions, then washed in PBS. Nissl Stain Kit (Solarbio Beijing, China) was used for Nissl staining. The Nissl bodies in the hippocampus CA1 and DG regions and cerebral cortex were examined under an optical microscope (Leica Microsystems Ltd., Wetzlar, Germany). Quantitative analysis of the Nissl body was conducted using the previous methods (Liu et al., 2023).

#### 2.1.6 Immunofluorescence staining

After deparrafinisation and rehydration, the brain sections were blocked with goat serum. After removal of the blocking solution, the sections were incubated with primary antibody against 5-methylcytosine (5-mC) diluted in blocking buffer. Unbound antibody was removed by PBS wash. The sections were then incubated with Alexa Fluor 488-conjugated goat anti-rabbit IgG. Nuclear counterstaining was performed with DAPI. Fluorescence imaging was conducted using an upright fluorescence microscope (Pi et al., 2022).

#### 2.1.7 Western blot

The hippocampus tissues from five mice in the same group were homogenized in RIPA lysis buffer with freshly added proteinase inhibitors. After centrifugation, the supernatant protein was measured using the BCA protein assay kit (Beyotime, China). The equal amounts of protein (30 μg) were separated on 10% NuPAGE Bis-Tris Protein gels, and transferred onto PVDF membrane (Millipore Trading Co. Ltd). To save PVDF membranes, we performed shearing on the internal reference protein and the target protein based on their molecular weights (Liu et al., 2018). The membranes were soaked in 5% skim milk, and then incubated with primary antibodies against P53 (Cell Signaling Technology, USA), P21, CDK4 and Nrf2 (Abcam, USA), respectively. After wash, the membranes were incubated with horseradish peroxidase-conjugated anti-rabbit or anti-mouse IgG secondary antibodies. The tests were repeated three times. Blots were visualized using chemiluminescence reagent BeyoECL Plus (Beyotime). Quantity One 1-D analysis software v4.52 (BioRad) was used for scanning the image and quantifying band intensity (Liu et al., 2018).

### 2.2 *C. elegans* experiment

#### 2.2.1 Animals

The wild-type N2 strain was obtained from Yue Cao (Changchun Institute of Applied Chemistry Academia of Sciences). CF1038 [*daf-16(mu86)*], EU1 [*skn-1(zu67)*] mutants were provided by Professor Huai-Rong Luo (Kunming Institute of Botany, Chinese Academy of Sciences). All strains were maintained and assayed at 20 °C on nematode growth medium (NGM) agar plates, which coated with *E. coli* OP50 (inactivate in a 65 °C water bath for 1 h) as a food source (Wang et al., 2024). The L4 larval stage serves as the starting point for all experimental analyses, which were consistent with our previous studies (Liu et al., 2026). For drug treatment, normal saline solution (Control), DMSO (0.1%) or FMN (0.37, 3.73, 37.30 μmol/L) was mixed with *E. coli* OP50(*Escherichia coli* OP50) on an NGM plate. The doses of FMN were selected based on previously published *in vitro* studies and by converting the measurements from experiments on *C. elegans* and cells (Dexter and Caldwell, 2012; Jin et al., 2025). A suspension was prepared and evenly spread onto the surface of NGM plates to form a bacterial lawn containing the drug. All of the following tests were repeated three times.

#### 2.2.2 Lifespan assays

On Day 0, at least 50 nematodes (synchronized L4 larvae) were transferred to NGM plates supplemented with inactivated *E. coli* OP50 (65 °C for 1 h). Floxuridine (FUDR, 12.5 mg/L) was used to suppress reproduction with or without FMN (0, 0.37, 3.73, 37.30 μmol/L) but does not replenish the bacterial lawn or remove metabolic waste. The nematodes were transferred to new NGM every other day to ensure the food was adequate. Survival was assessed daily using a touch-provocation assay. Nematodes displaying rigidity and absence of movement upon platinum wire stimulation were scored as deceased (Ouyang et al., 2026). *C. elegans* were excluded if they exhibited gonadal abnormalities, death, or larval hatching within adults. Lifespan assays were performed at the same time daily at 20 °C until all nematodes died. Data were analyzed by IBM SPSS software, independent biological replicates were pooled for analysis. We generated Kaplan-Meier survival curves, group differences were analyzed by using the log-rank test, lifespan assays were repeated at least three times.

#### 2.2.3 Thermotolerance assays

Age-synchronized L4 larvae were bred on NGM agar plates with or without FMN (37.3 μmol/L) for 72 h at 20 °C (n ≥ 50 for each plate) (Liu et al., 2026; Wang et al., 2024). Subsequently, adult nematodes were transferred to fresh NGM supplemented with *E. coli* OP50 (NGM was pre-heated at 35 °C for 2 h). Heat stress was conducted at 35 °C for 2 h, then the nematodes were continuously fed at 20 °C. The number of dead individuals was recorded at the same time daily until all nematodes died.

#### 2.2.4 Paraquat assays

To test the effect of FMN on survival under oxidative stress. At least 50 synchronized L4 were added to each NGM agar plate containing 12.5 mg/L FUDR, *E. coli* OP50 (200 μL), and 37.3 μmol/L FMN at 20 °C. After 72 h, the worms were transferred to fresh NGM and 2 mM paraquat was added. The number of dead individuals was recorded at the same time daily until all nematodes died (Liu et al., 2026).

#### 2.2.5 Mobility assays

Body bend frequency is a quantitative measure of spontaneous movement. We compared the mobility of L4 nematodes in a Control group (n = 30) to a DNLA group (37.3 μmol/L, n = 30). We examined and counted the number of body bends performed over 20 s at the same time daily until the 13th day (Zeidan et al., 2025).

#### 2.2.6 Progeny production assays

To fertility of *C. elegans*, 10 age-synchronized L4 larvae growing on NGM plates were supplemented with or without FMN (37.3 μmol/L) and were transferred to fresh plates daily until reproduction finished. The progeny of each nematode was counted after 7 days.

#### 2.2.7 Lipofuscin detection

We randomly selected 30 individuals of L4 larvae growing on NGM plates were supplemented with or without FMN (37.3 μmol/L) on the 10th days and scored their intestinal lipofuscin levels. First, we anesthetized with levamisole, then acquired a fluorescent image with an Olympus fluorescent microscope (BX41-32P02-FLB3). The excitation wavelength ranged from 360 to 370 nm, the emission wavelength ranged from 420 to 460 nm,and the exposure time was set to 100–500 m. The image acquisition parameters were set consistently across all groups. Image Pro Plus 6.0 software was used for statistical analysis to quantify the autofluorescence intensity of lipofuscin in the images. The intestinal region (from the pharynx to the rectum) was selected as the region of interest (ROI), avoiding intestinal contents. The corrected total fluorescence intensity (Corrected Total IntDen) was calculated as: ROI IntDen - (ROI area × mean background gray value). Fluorescence intensities were then normalized to the Control group to obtain relative lipofuscin levels (Hou et al., 2026).

#### 2.2.8 Total SOD activity

We compared ≥300 L4 larvae control individuals to ≥300 L4 larvae in the FMN (37.3 μmol/L) group. Total nematode protein was quantified using a BCA protein quantification kit (Shanghai Generay, China), total SOD activity was detected by using a total superoxide dismutase kit (Nan Jing Jian Cheng Bioengineering Institute, China). Three biological replicates were performed in this experiment.

#### 2.2.9 RT-qPCR *assay*

Approximately 1000 synchronized wild-type L4 larvae were transferred to NGM plates (10 cm diameter) with or without 37.3 μmol/L FMN and cultured at 20 °C for 72 h. Total RNA was extracted using Trizol (Takara, Japan) and a Reverse Transcription Kit (Applied Taraka) was used to synthesize cDNA. The PCR reaction system was 15 μL (7.5 μL SYBR super mix, 0.5 μL of 10 μM of each primer, 3 μL of cDNA, and 4 μL of DEPC water). The following conditions were used for real-time PCR: 95 °C for 10 min, then 95 °C for 15 s, and 60 °C for 1 min in 40 cycles. The gene expression levels were determined by the quantification cycle values (Cq), followed by the standard 2^−ΔΔCt^ method (Liu et al., 2026). Gene expression levels of *act-1* were used as the internal control. Each group was normalized to the Control group, which was set at 100%. Primer sequences were as follows (Table 1).

**TABLE 1:** RT-qPCR primer sequences.

Gene	Forward	Reverse
*act-1*	GCT​GAT​CGT​ATG​CAG​AAG​GAA​A	GTT​GGA​AGG​TGG​AGA​GGG​AAG
*daf-16*	CGGGTGCCTATGGAAACT	TTGGAAGAGCCGATGAAG
*sod-3*	AGC​ATC​ATG​CCA​CCT​ACG​TGA	CAC​CAC​CAT​TGA​ATT​TCA​GCG
*Sir-2.1*	GGC​AGA​AAC​TCA​CAA​ACT​ACA​A	GAA​GTC​GTG​CAT​AAA​TAC​CAT​C
*skn-1*	AGT​GTC​GGC​GTT​CCA​GAT​TTC	GTC​GAC​GAA​TCT​TGC​GAA​TCA

#### 2.2.10 Mutant nematode lifespan

To verify the role of *daf-16* and *skn-1* in FMN protection, CF1038 [*daf-16(mu86)*] and EU1 [*skn-1(zu67)*] mutants were cultured to L4. Well grown nematodes were transferred to culture plates and divided into a Control group and a 37.3 μmol/L FMN treatment group (n = 30 per group). The worms were cultured at a constant temperature of 20 °C, and this time point was recorded as day 0 of the lifespan assay. Nematode deaths were recorded daily until all individuals died. In short, we generated Kaplan-Meier survival curves and used the log-rank test to assess group differences.

### 2.3 Statistics

Experimental data were analyzed by using SPSS29.0 (IBMCorp, Armonk, NY, USA, Version 29.0) statistical software. Results are presented as mean ± standard deviation (mean ± SD). Unless otherwise specified, statistical significance was evaluated by using one way ANOVA, followed by Bonferroni test or Dunnett’s T3 test. We used a threshold of *p* < 0.05 to determine statistical significance.

## 3 Results

### 3.1 Effects of FMN on cognition, anxiety and motor endurance in SAMP8 mice

The experimental design is shown below (Figure 1A). The short-term spatial working memory was evaluated by Y maze test (Figure 1B) and NOR test (Figure 1C). Compared with the age-matched SAMR1 mice, the short-term spatial working memory of 9-month-old SAMP8 mice was significantly decreased (p < 0.05). Fortunately, after 5 months of FMN treatment, short-term spatial working memory improved in a dose-dependent manner (especially at the doses of 8 mg/kg and 16 mg/kg, Figure 1B). At the same time, compared with the SAMR1 group, the NOR test found the explore new objects time was significantly reduced in SAMP8 group (p < 0.05). After FMN treatment, the percentage of explore time significantly increased (especially at the dose of 16 mg/kg, Figure 1C). These results indicated that FMN improved the cognitive deficits of SAMP8 mice.

**FIGURE 1:**
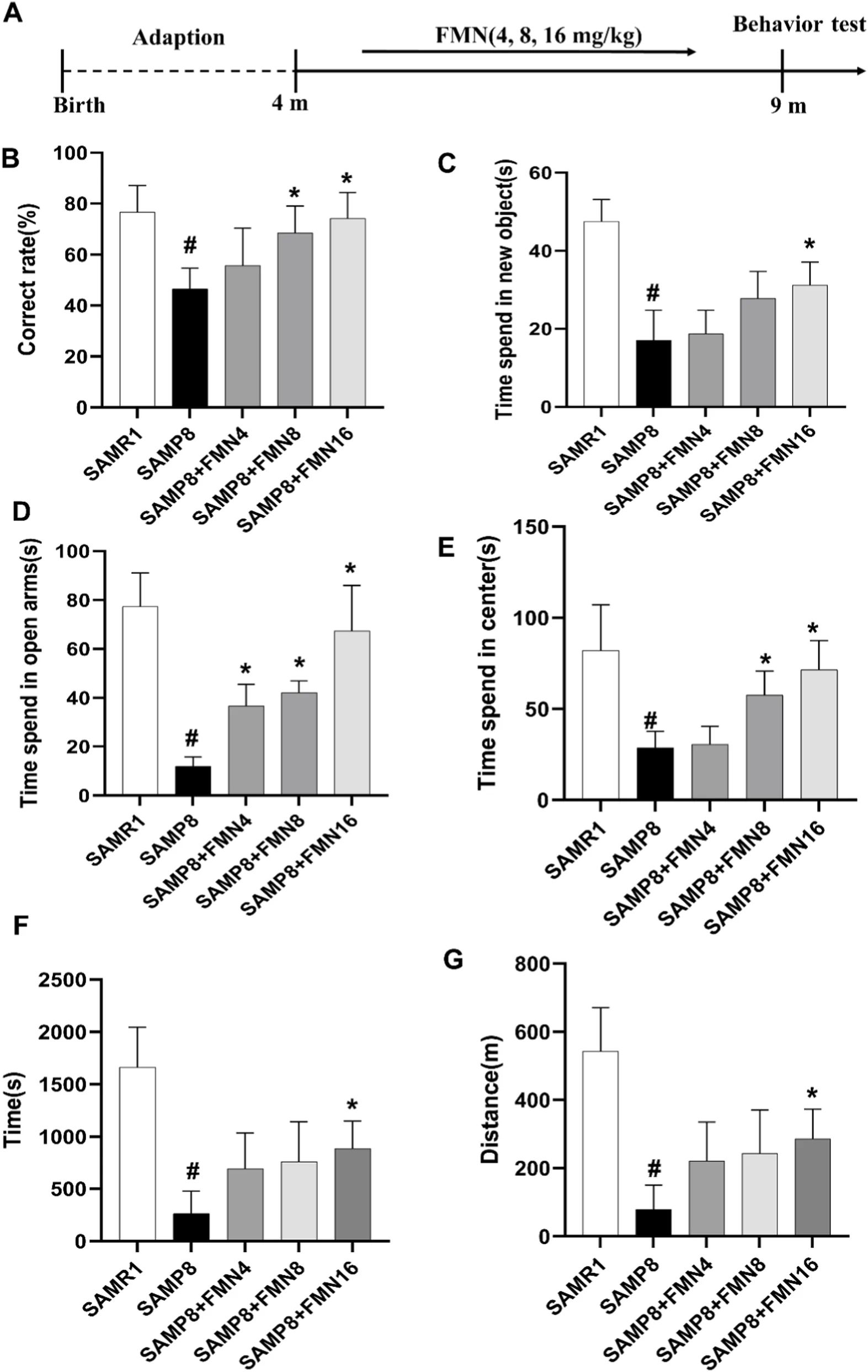
Effects of FMN on Cognition, Anxiety and Motor Endurance in SAMP8 Mice. **(A)** Schematic drawing of the experimental design. **(B)** The correct rate of Y Maze test spontaneous alternation. **(C)** The percentage of time spent in exploring the new object. **(D)** The number of entries into the open arms. **(E)** The activity time spent in central region. **(F)** The movement time (s); **(G)** The movement distance (m) (Mean ± SD, n = 7–8), #*p* < 0.05 vs. SAMR1 group, *p < 0.05 vs. SAMP8 group.

Anxiolytic-like behaviors were evaluated by EPM (Figure 1D) and OFT test (Figure 1E). The EPM test revealed that the SAMP8 mice exhibited anxiety-like behaviors, as evidenced by a significant decrease in the number of entries with open arms. Fortunately, FMN increased the number of entries into the open arms (Figure 1D). The OFT demonstrated that the time of SAMP8 mice was significantly reduced in the central region. After FMN treatment, the time spent in the central area shows a dose-dependent increase (Figure 1E). These results clearly indicated that FMN improved the anxiety-like behaviors of SAMP8 mice (p < 0.05).

We next analyzed the effect of FMN on exercise performance SAMR1 and SAMP8 mice were subjected to treadmill exercise test until severe fatigue. Regarding exercise time, SAMR1 mice were able to exercise longer than SAMP8 mice before exhaustion (Figure 1F). Regarding the running distance, SAMR1 mice could run longer than SAMP8 mice (Figure 1G). After 5 months of FMN treatment, the duration and distance of movement in SAMP8 mice was increased. These results indicate that long-term FMN administration elevated endurance capacity in mice.

### 3.2 FMN attenuates neuronal damage in the hippocampus and cortex in SAMP8 mice

Neuronal morphology in CA1 and DG regions of the hippocampus and cerebral cortex were examined by Nissl staining. In the SAMR1 group, granule neurons exhibited round nuclei, which were located in the center of the perikaryon and surrounded by a pale cytoplasm. A significant loss of Nissl-stained neurons and vacuoles, as well as a large number of pyknotic neurons were observed in SAMP8 mice. After treatment with three doses of FMN, Nissl-stained neurons in the normal form in the CA1 and DG region of the hippocampus and cerebral cortex were increased, especially in the high-dose group (Figure 2, p < 0.05).

**FIGURE 2:**
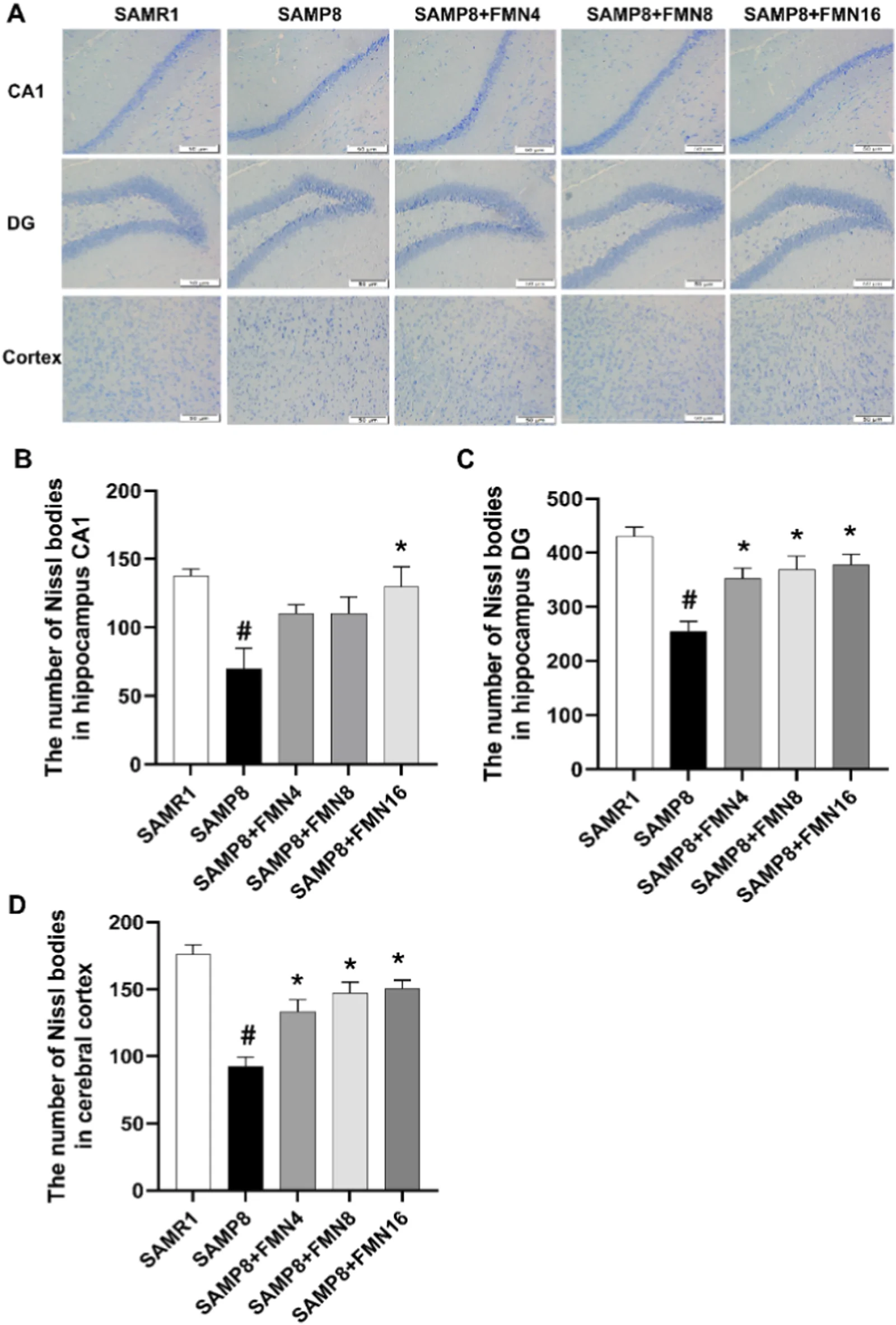
Effect of FMN on neuron morphology change in SAMP8 mice. **(A)** Representative images showing Nissl bodies in CA1 and DG regions of hippocampus and cerebral cortex (magnification ×200, scalebar = 50 μm). **(B)** Quantitation of Nissl-stained neurons in CA1 regions of hippocampus. **(C)** Quantitation of Nissl-stained neurons in DG regions of hippocampus. **(D)** Quantitation of Nissl-stained neurons in cerebral cortex (Mean ± SD, n = 5), #p < 0.05 vs. SAMR1 group; *p < 0.05 vs. SAMP8 group.

### 3.3 FMN increased 5-mC level in the hippocampus and cortex of SAMP8 mice

DNA methylation level can be directly demonstrated by the expression of 5-mC. Compared with the SAMR1 group, the level of 5-mC in the CA1 and DG region of the hippocampus and cerebral cortex of the SAMP8 group was significantly lower. Compared with the SAMP8 group, the level of 5-mC in the CA1 and DG region of the hippocampus and cerebral cortex was significantly increased after FMN treatment. The 5-mC immunofluorescence results suggest that FMN can increase the level of 5-mC in the hippocampus CA1 and DG region and cortex of the SAMP8 mice. The corresponding DAPI or phalloidin staining were provided in the (Figure 3) Supplementary Figure S1; Supplementary Figure S2; Supplementary Figure S3.

**FIGURE 3:**
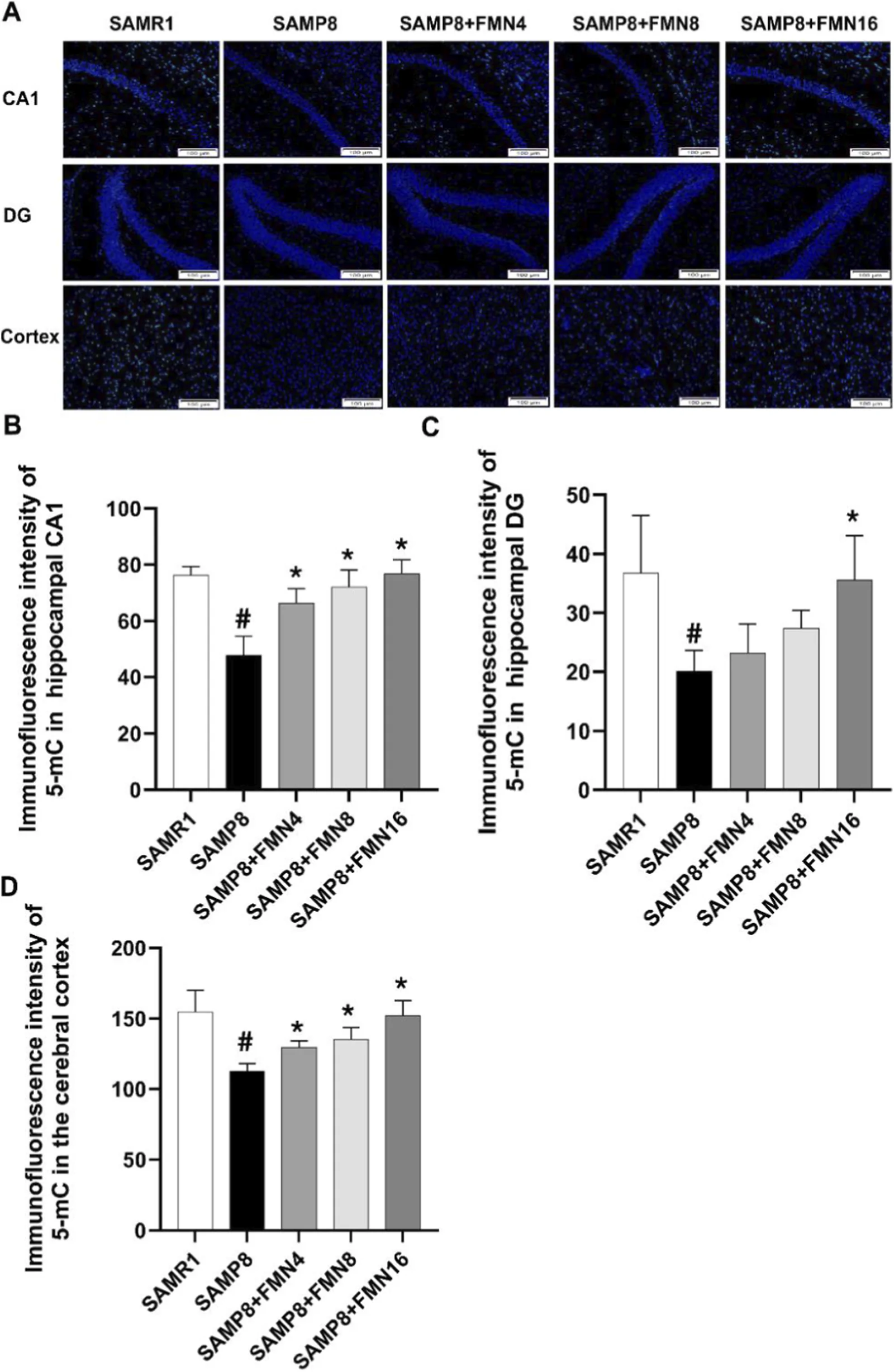
Effect of FMN on 5-mC level in the hippocampus and cortex of SAMP8 mice. **(A)** Representative images showing 5-mC immunofluorescence in CA1 and DG regions of hippocampus and cerebral cortex (scalebar = 100 μm). **(B)** Quantitation of 5-mC immunofluorescence in CA1 regions of hippocampus. **(C)** Quantitation of 5-mC immunofluorescence in DG regions of hippocampus. **(D)** Quantitation of 5-mC immunofluorescence in cerebral cortex (Mean ± SD, n = 5), #*p* < 0.05 vs. SAMR1 group; *p < 0.05 vs. SAMP8 group.

### 3.4 FMN regulated the expression of P53, P21, CDK4 and Nrf2 in the hippocampus of SAMP8 mice

P53, P21 and CDK4 are the core molecules regulating the cell cycle, and the three of them jointly regulate aging through a tight signaling network (Crozier et al., 2023). Figure 4 shows significant alterations in the levels of P53, P21, CDK4 in hippocampus of SAMP8 mice compared with age-matched SAMR1 mice (p < 0.05). FMN treatment for 5 months reduced the expression of P53 and P21, as well as increased the expression of CDK4. Moreover, FMN treatment also increased the expression of Nrf2 in hippocampus of SAMP8 mice. These results indicate that FMN plays a role in regulating the cell cycle and increasing the expression of Nrf2.

**FIGURE 4:**
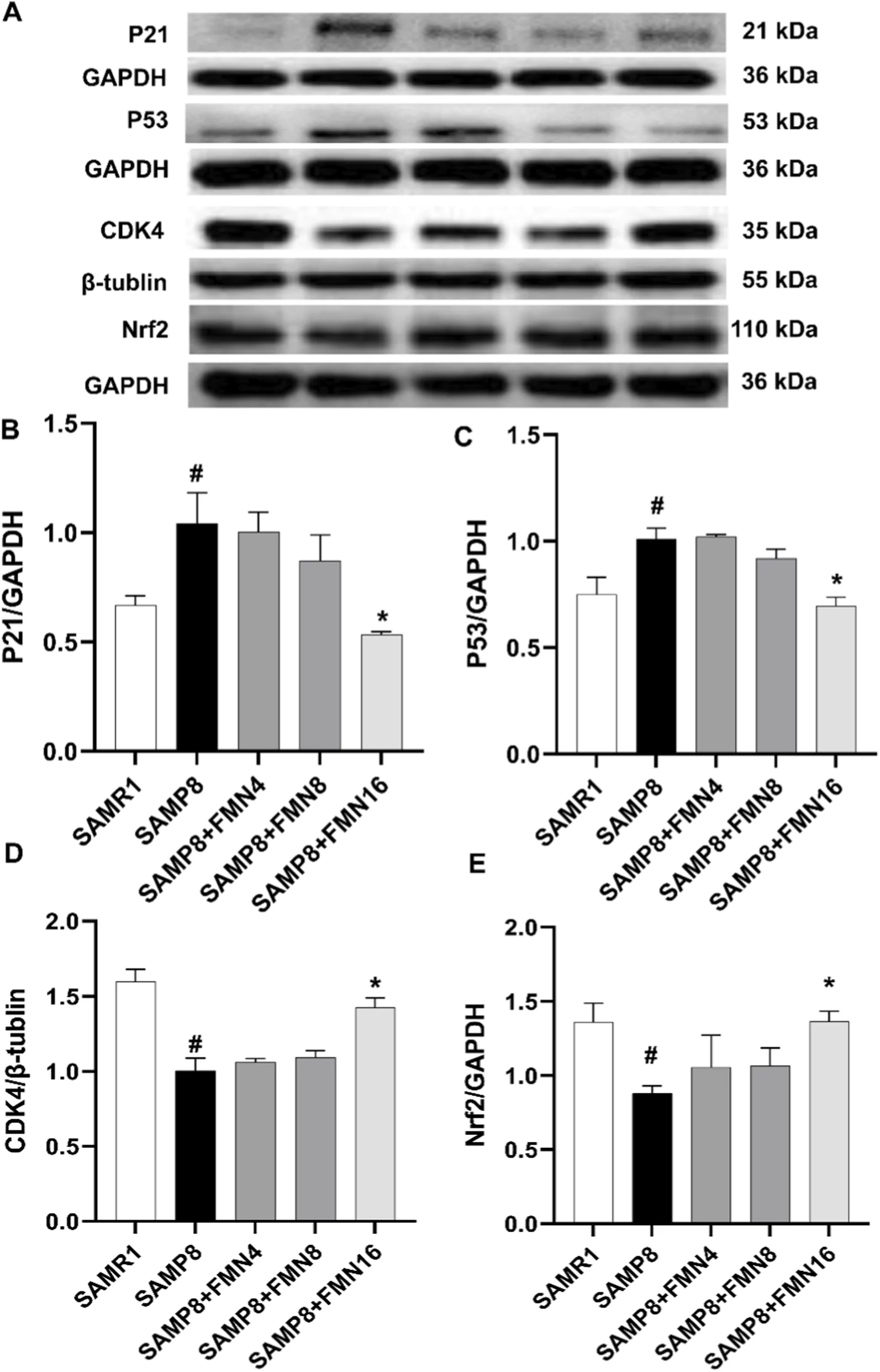
Effect of FMN on the expression of P53, P21, CDK4 and Nrf2 in the hippocampus of SAMP8 mice. **(A)** Expression of P53, P21, CDK4 and Nrf2 proteins in the hippocampus. **(B)** Quantitation of P21 level. **(C)** Quantitation of P53 level. **(D)** Quantitation of CDK4 level. **(E)** Quantitation of Nrf2 level (Mean ± SD, n = 5), #p < 0.05 vs. SAMR1 group; *p < 0.05 vs. SAMP8 group.

### 3.5 FMN prolongs the lifespan of *C. elegans* under normal conditions, thermal stress and oxidative stress

Compared with the Control group, FMN dose-dependent prolonged the lifespan of *C. elegans*, with the dose of 37.30 μmol/L had the most significant effect on extending lifespan. Therefore, FMN at the dose of 37.30 μmol/L was chosen for the subsequent research (Figures 5A,B). Moreover, under a 35 °C thermal stress (Figure 5C), the average survival was increased in 37.30 μmol/L FMN pre-treated group compared to the Control group (p < 0.05), and the lifespan increased (Figure 5D). Under paraquat induced oxidative stress conditions, the 37.30 μmol/L FMN group shifted the survival curve to the right (Figure 5E). Compared with the Control group, FMN can significantly prolong the lifespan of nematodes under paraquat induced stress conditions (Figure 5F, p < 0.01). These data showed that FMN prolongs *C. elegans* lifespan under normal conditions, thermal stress and oxidative stress conditions.

**FIGURE 5:**
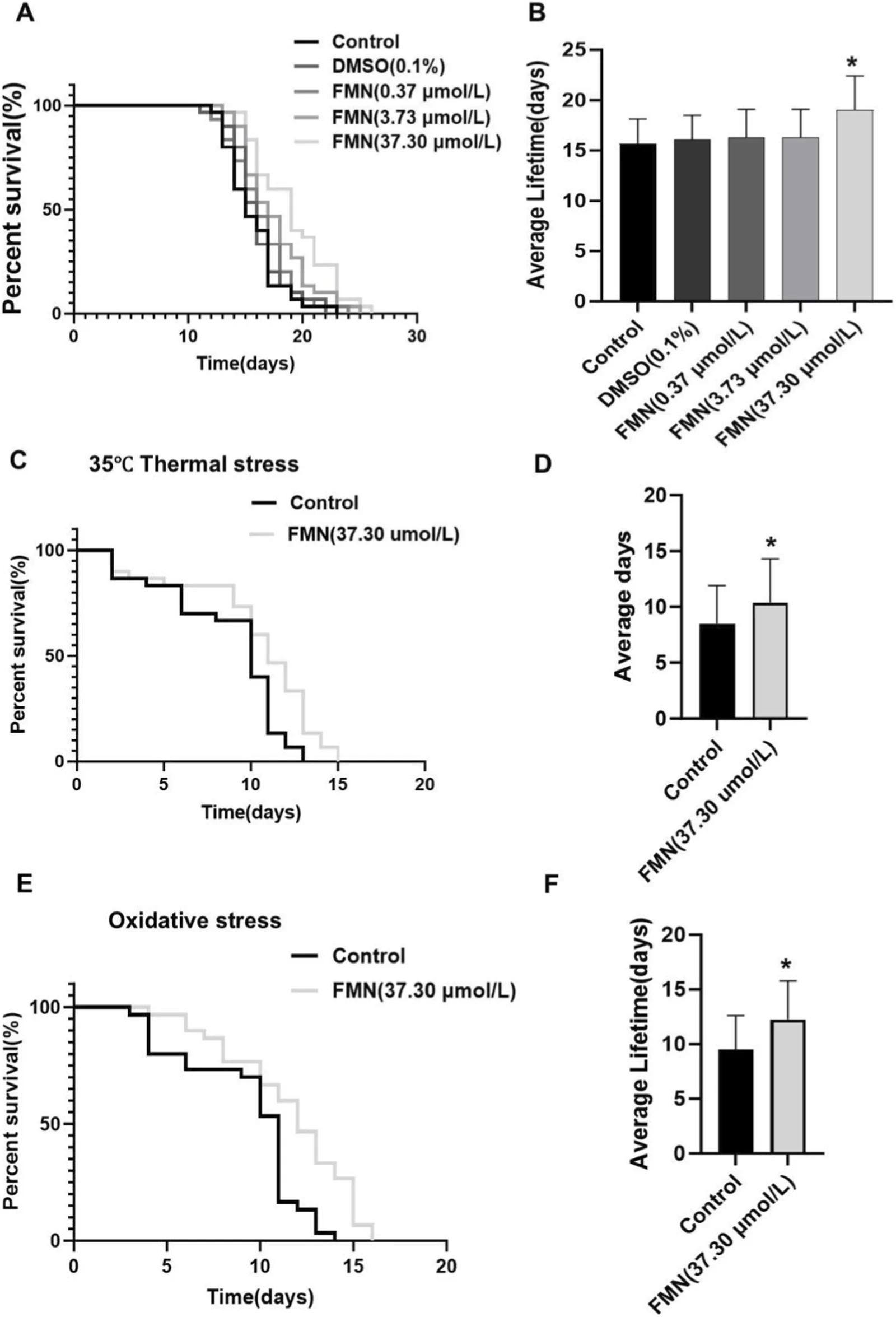
Effect of FMN on the lifespan of *C. elegans* under different conditions. **(A)** The survival curve and average life of *C. elegans* under normal conditions. **(B)** Lifespan of *C. elegans* under different concentrations of FMN at normal conditions. **(C)** The survival curve of *C. elegans* under thermal stress. **(D)** Effects of FMN on longevity of *C. elegans* under thermal stress. **(E)** The survival curve of *C. elegans* under oxidative stress. **(F)** Effects of FMN on longevity of *C. elegans* under oxidative stress (Mean ± SD, n = 30), *p < 0.05 vs. Control group.

### 3.6 Effects of FMN on lifespan and stress resistance in *C. elegans*

The effects of FMN on physiological parameters in *C. elegans* were assessed with respect to locomotor function and reproductive capacity. FMN exerted a time-dependent modulatory effect on locomotion. Specifically, the frequency of the nematode’s body curvature remained unchanged within 20 s compared to Control group on day 6 post-treatment; however, statistically significant increases were detected on days 9 and 13 in *C. elegans* exposed to FMN at a concentration of 37.30 μmol/L. No significant alterations were observed in reproductive output: neither the daily egg-laying rate nor the total progeny count differed significantly between FMN-treated and untreated Control group (Figures 6B,C). Lipofuscin, a well-established biomarker of cellular aging, accumulates progressively in the intestinal tissues of *C. elegans* during aging, emitting characteristic autofluorescence detectable by upright fluorescence microscopy. Quantitative assessment revealed that nematodes treated with FMN at 37.30 μmol/L exhibited significantly reduced intestinal lipofuscin autofluorescence intensity relative to the untreated Control group (Figures 6D,E). Concurrently, T-SOD activity was significantly elevated in the FMN-treated group compared with the Control (Figure 6F). These findings collectively suggest that FMN supplementation may attenuate lipofuscin accumulation and age-associated oxidative damage in *C.elegans*, potentially contributing to lifespan extension *via* enhancement of endogenous antioxidant defense capacity.

**FIGURE 6:**
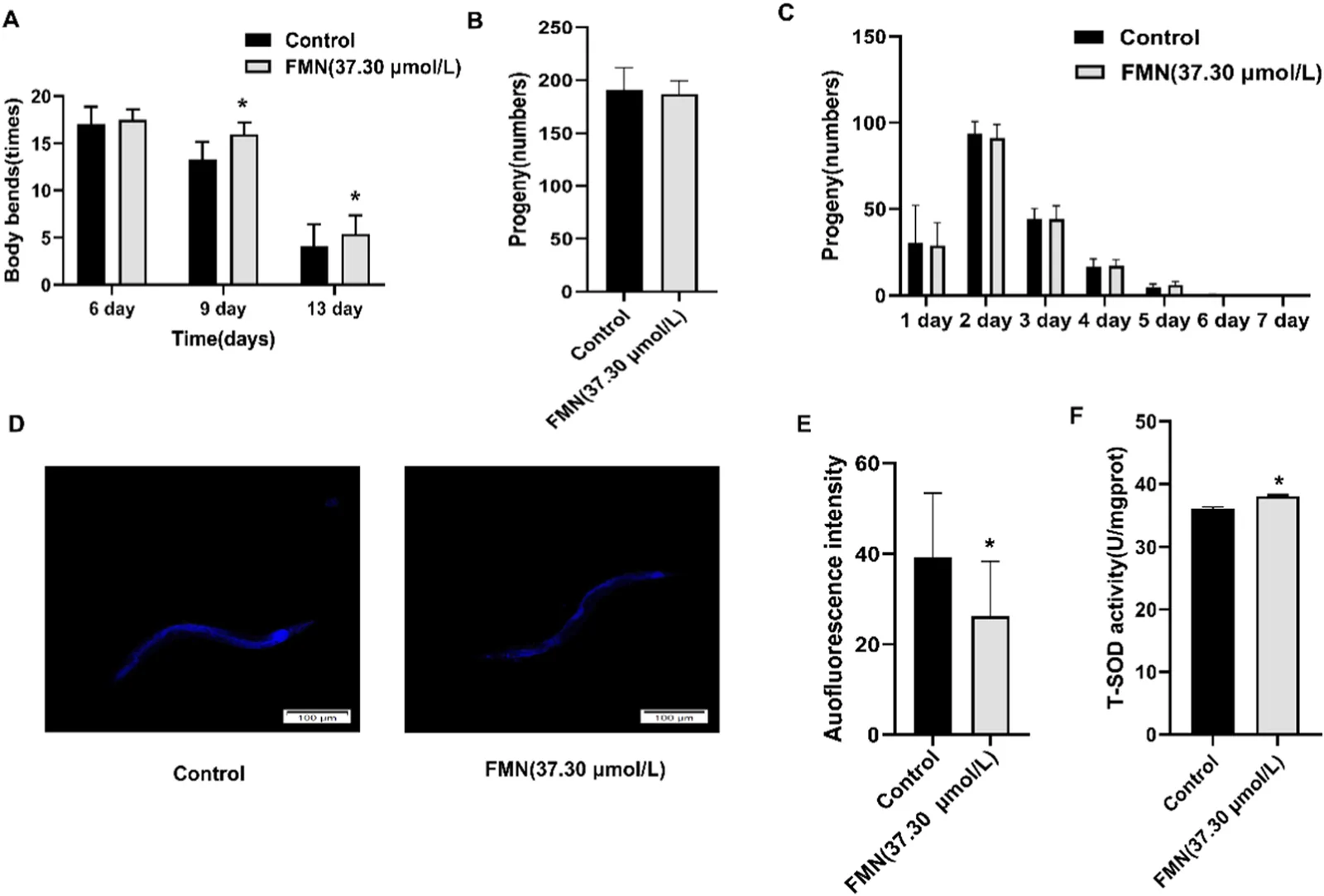
Effects of FMN on lifespan and stress resistance in *C. elegans*. **(A)** The number of body bends on the sixth, ninth, and 13th days of cultivation. **(B)** Total egg production. **(C)** Daily egg production during the breeding period. **(D,E)** Effects of FMN on lipofuscin accumulation in nematodes (Scale = 100 um). **(F)** Quantitative analysis of T-SOD activity in *C. elegans* (Mean ± SD, n = 10), *p < 0.05 vs. Control group.

### 3.7 Effects of FMN on gene expression in *C. elegans*

RT-qPCR showed that compared with the Control group, the FMN (37.30 μmol/L) group exhibited increased mRNA expression of *skn-1, sir2.1, daf-16* and *sod-3* in *C. elegans*. The results suggest that FMN may delay the aging of the worms by regulating mRNA expression of *skn-1, sir2.1, daf-16* and *sod-3* (Figures 7A–D). Compared with the Control group, after being treated with 37.30 μmol/L FMN, there were no significant differences in the survival curves and average survival days of the EU1[*skn-1(zu67)*] and CF1038[*daf-16(mu86)*] mutant strains (Figures 7E–H). Therefore, the effect of FMN on promoting longevity and stress resistance is associated with the *daf-16* and *skn-1* genes.

**FIGURE 7:**
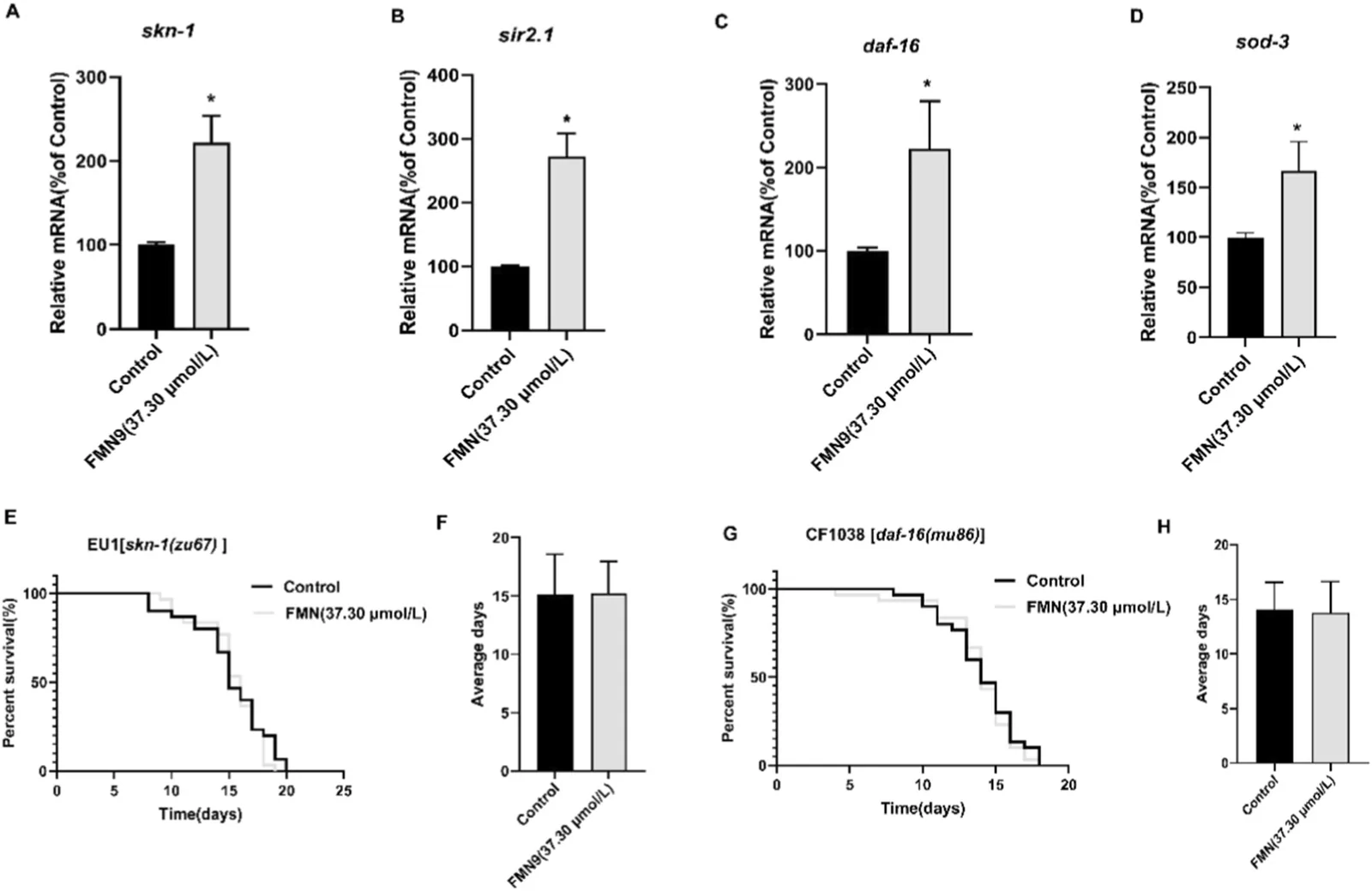
Real-time PCR results. **(A)** The mRNA expression *of skn-1*. **(B)** The mRNA expression *of sir2.1.* **(C)** The mRNA expression *of daf-16.* **(D)** The mRNA expression *of sod-3*. (mean ± SD, n = 6), **(E)** Survival curve of CF1038 [*daf-16(mu86)*] mutant strain. **(F)** Average survival days of CF1038 [*daf-16(mu86)*] mutant strain. **(G)** Survival curve of EU1[*skn-1(zu67)*] mutant strain. **(H)** Average survival days of EU1[*skn-1(zu67)*] mutant strain (Mean ± SD, n = 30). *p < 0.05 vs. Control group.

## 4 Discussion

The present study revealed that: (1) FMN dose-dependently ameliorated cognitive deficits, anxiety-like behaviors, and reduced motor endurance in SAMP8 mice; (2) FMN alleviated neuronal damage in the hippocampus and cortex and increased 5-mC level; (3) FMN downregulated the pro-aging factors P53 and P21, and upregulated CDK4 and Nrf2; (4) In *C. elegans*, FMN extended lifespan, enhanced resistance to thermal and oxidative stress, reduced lipofuscin accumulation, and acted through the DAF-16 and SKN-1 pathways dependency.

The SAMP8 mouse exhibits significant learning and memory impairment, anxiety-like behavior, and neuronal damage at 9 months of age, accompanied by abnormal DNA methylation patterns (Griñán-Ferré et al., 2024; Liu et al., 2020b). In the present study, 9-month-old SAMP8 mice showed significantly decreased spontaneous alternation rates in the Y-maze and reduced exploration time in the NOR test, as well as pronounced anxiety-like behavior in the elevated plus maze and open field test, consistent with the typical phenotype of SAMP8 mice (Griñán-Ferré et al., 2024). Long-term (5 months) treatment with FMN significantly improved these behavioral indicators, with the most pronounced effects observed at the dose of 16 mg/kg. Nissl staining further demonstrated that FMN reversed the reduction of Nissl bodies in the hippocampal CA1 and DG regions and the cerebral cortex of SAMP8 mice. These findings collectively confirm the potential of FMN as a neuroprotective agent in ameliorating age-related cognitive dysfunction, which is consistent with our previous studies (Liu et al., 2024).

DNA methylation is a key epigenetic mechanism regulating genomic stability and gene expression, undergoing characteristic global hypomethylation during aging (Field et al., 2018). In this study, 5-mC immunofluorescence staining revealed that 5-mC level in the hippocampus and cortex of SAMP8 mice were significantly lower than those in the SAMR1 Control group, while FMN treatment significantly restored 5-mC level. This finding suggests that age-related 5-mC level may be an epigenetic basis for cognitive dysfunction in SAMP8 mice, and that FMN exerts neuroprotective effects by reversing this abnormal hypomethylation. Database analysis indicates that FMN can regulate TP53 activity through methylation enrichment mechanisms, providing a possible molecular pathway for FMN’s reversal of methylation abnormalities (Aliya et al., 2023). Notably, changes in DNA methylation during aging often precede clinical symptoms; therefore, FMN’s restoration of 5-mC level may represent not only a therapeutic effect but also its potential for early intervention in the aging process.

The P53/P21 signaling pathway is a central node regulating cell cycle arrest and cellular senescence (Yu et al., 2025b). P53, the “guardian of the genome”, is activated under stress conditions such as DNA damage and telomere dysfunction, subsequently inducing expression of its downstream target gene P21 (Engeland, 2022). P21 inhibits CDK-cyclin complex activity, leading to G1/S phase cell cycle arrest and ultimately promoting cellular senescence (Henri et al., 2020). In this study, we found that P53 and P21 protein expression levels were significantly elevated in the hippocampus of SAMP8 mice, while CDK4 expression was significantly decreased; FMN treatment reduced P53 and P21 expression and restored CDK4 levels. Interestingly, FMN exhibits a complex bidirectional regulatory relationship with the P53 signaling pathway. In various cancer models, FMN induces cancer cell cycle arrest and apoptosis by upregulating P53 and P21 expression (Sharma and Kabra, 2025; Zhang et al., 2019). However, in the present study, FMN downregulated P53 and P21 expression in the context of normal aging. This discrepancy may be attributed to tissue specificity, cell state (proliferative vs. terminally differentiated), and the “threshold” of P53 signaling: in cancer cells, moderate upregulation of P53 is sufficient to trigger apoptosis; whereas in aging neurons, persistent P53 activation instead promotes cellular senescence and functional decline, and FMN restores neuronal vitality by inhibiting such excessive P53 activation. Furthermore, the CTD database indicates that FMN can regulate TP53 activity through methylation enrichment pathways (Aliya et al., 2023), which aligns with our finding that FMN upregulates 5-mC level, suggesting that FMN may indirectly regulate P53 expression by reversing age-related 5-mC level.

Nrf2 is a core transcription factor regulating the endogenous antioxidant defense system, and its activity significantly declines during aging, leading to increased oxidative stress damage (Vukojević et al., 2026). In this study, Nrf2 protein expression levels were significantly reduced in the hippocampus of SAMP8 mice, while FMN treatment significantly restored Nrf2 expression. Numerous studies have confirmed that FMN exerts antioxidant effects by regulating Nrf2 (Huang et al., 2017; Sugimoto et al., 2021; Wang et al., 2024). These lines of evidence indicate that activation of Nrf2-mediated antioxidant defense might be a core mechanism underlying FMN’s protective effects.

*C. elegans* is a classic model organism for studying aging and longevity, characterized by a short lifespan (approximately 2–3 weeks), genetic tractability, and highly conserved aging-related signaling pathways (Liu et al., 2026; Yasuda, 2025). Using wild-type N2 nematodes, we found that FMN dose-dependently extended the lifespan of *C. elegans* (with 37.30 μmol/L showing the best effect) and significantly enhanced resistance to thermal stress (35 °C) and oxidative stress (paraquat-induced). FMN treatment also improved locomotion (increased body bend frequency on days 9 and 13), reduced lipofuscin accumulation (a marker of cellular senescence), and increased total SOD activity, without significantly affecting reproductive capacity–indicating that FMN extends lifespan not through the “metabolic cost” mechanism of inhibiting reproduction. RT-qPCR results showed that FMN significantly upregulated the mRNA expression of *skn-1*, *sir-2.1*, *daf-16*, and *sod-3*. Mutant validation further demonstrated that in *daf-16* and *skn-1* deficient mutant nematodes, FMN’s lifespan-extending effect was completely abolished, confirming that DAF-16 (the mammalian FOXO homolog) and SKN-1 (the mammalian Nrf2 homolog) are essential signaling nodes for FMN’s longevity effects (Blackwell et al., 2015; Kenyon, 2010). DAF-16 and SKN-1 represent two classic and interconnected longevity pathways in *C. elegans*, corresponding to the mammalian FOXO and Nrf2 signaling axes, respectively. FMN’s simultaneous activation of both pathways reflects the unique advantage of natural products in achieving “multi-target synergistic” regulation of the aging network.

In this study, we observed effects of FMN in two evolutionarily distant species–*C. elegans* and mice–with highly conserved molecular mechanisms: in *C. elegans*, FMN acts through the DAF-16 (FOXO) and SKN-1 (Nrf2) pathways to extend lifespan; in mice, FMN upregulates Nrf2 expression and regulates the P53/P21/CDK4 aging signaling axis. The SAMP8 mouse is a reliable model for studying age-related cognitive decline, with pathogenesis involving abnormal expression of factors, increased oxidative stress, and neuroinflammation (Liu et al., 2020b; Morley et al., 2012). Integrating these findings, we can delineate a molecular map of FMN’s effects: FMN (1) activates the Nrf2/SKN-1 antioxidant defense system, reducing cumulative oxidative damage during aging; (2) regulates the P53/P21/CDK4 cell cycle/senescence signaling axis to inhibit aberrant cellular senescence; and (3) increases age-related 5-mC level, and restores genome-wide epigenetic regulatory homeostasis. The coordinated action of these three possible pathways ultimately achieves comprehensive effects of delaying aging, improving cognitive function, and enhancing stress resistance across different species.

This study has several limitations. First, the findings related to the DAF-16/FOXO pathway observed in *C. elegans* lack direct validation in mammals. Although Nrf2 has been confirmed as a target of FMN in mice, the role of the FOXO pathway in the effects of FMN requires further confirmation through gene knockout or inhibitor experiments in mammalian systems. Second, the precise molecular mechanism by which FMN reverses DNA hypomethylation remains unclear–whether FMN directly inhibits DNA methyltransferase activity or indirectly influences methylation patterns through upstream signaling pathways requires further investigation using techniques such as methylome sequencing and DNMT activity assays. Third, while this study evaluated FMN’s effects on health and lifespan in *C. elegans*, the intervention in mice started at 4 months of age (equivalent to human young adulthood) and continued until 9 months of age (transition from middle to old age). Whether FMN is equally effective in older mice (e.g., 12–15 months old) remains to be determined. Fourth, positive drugs should be used in further research.

## 5 Conclusion

In SAMP8 mice, FMN improves cognitive function, alleviates anxiety-like behavior, and enhances motor endurance, while protecting against neuronal damage in the hippocampus and cortex. Mechanistically, FMN reverses age-associated 5-mC level, downregulates the pro-aging factors P53 and P21, upregulates CDK4, and activates the Nrf2 antioxidant pathway. In *C. elegans*, FMN extends lifespan, increases resistance to thermal and oxidative stress, reduces lipofuscin accumulation, and improves locomotion, with these effects requiring the DAF-16 and SKN-1 signaling pathways dependency (Figure 8). Collectively, these findings establish that FMN holds promise as a candidate natural product for the development of healthy aging interventions.

**FIGURE 8:**
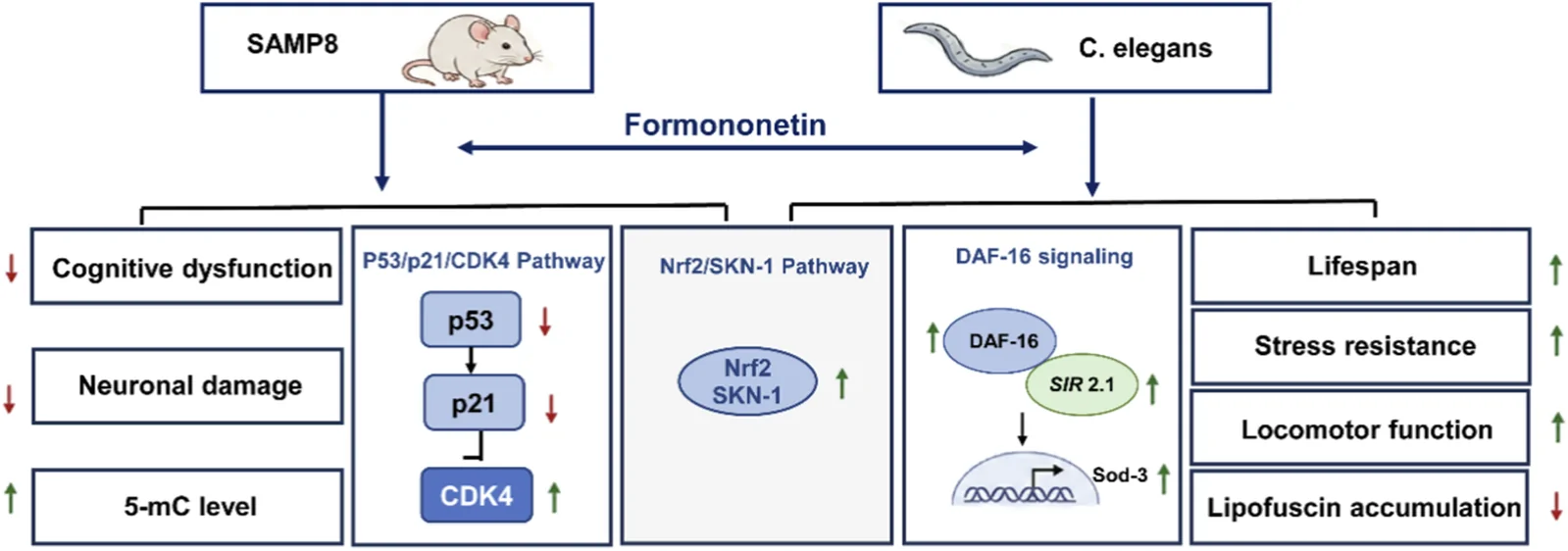
Schematic illustration of the mechanisms of FMN.

## Data Availability

The original contributions presented in the study are included in the article/Supplementary Material, further inquiries can be directed to the corresponding author.
