# Supplementary material for: Formononetin improved healthspan via the P53/P21/CDK4 pathway in SAMP8 mice and DAF-16/SKN-1 signaling pathway in C.elegans
Source: Front Pharmacol. 2026 Sep 23;17:1918524. doi: 10.3389/fphar.2026.1918524 (PMC13642585; doi:10.3389/fphar.2026.1918524)
Supplement: Supplementary file 1 [file Supplementaryfile1.docx]

**Supplementary Materials**

**Supplementary Figure 1.** Representative images showing 5-mC immunofluorescence in CA1 regions of hippocampus (scalebar =100 μm).

**Supplementary Figure 2.** Representative images showing 5-mC immunofluorescence in DG regions of hippocampus (scalebar =100 μm).

**Supplementary Figure 3.** Representative images showing 5-mC immunofluorescence in cortex (scalebar =100 μm).
